# ISLES 2022: A multi-center magnetic resonance imaging stroke lesion segmentation dataset

**DOI:** 10.1038/s41597-022-01875-5

**Published:** 2022-12-10

**Authors:** Moritz R. Hernandez Petzsche, Ezequiel de la Rosa, Uta Hanning, Roland Wiest, Waldo Valenzuela, Mauricio Reyes, Maria Meyer, Sook-Lei Liew, Florian Kofler, Ivan Ezhov, David Robben, Alexandre Hutton, Tassilo Friedrich, Teresa Zarth, Johannes Bürkle, The Anh Baran, Björn Menze, Gabriel Broocks, Lukas Meyer, Claus Zimmer, Tobias Boeckh-Behrens, Maria Berndt, Benno Ikenberg, Benedikt Wiestler, Jan S. Kirschke

**Affiliations:** 1 Department of Diagnostic and Interventional Neuroradiology, Klinikum rechts der Isar, School of Medicine, Technical University of Munich, Munich, Germany; 2 icometrix, Leuven, Belgium; 3 Department of Informatics, Technical University of Munich, Munich, Germany; 4 Department of Diagnostic and Interventional Neuroradiology, University Medical Center Hamburg-Eppendorf, Hamburg, Germany; 5 Institute of Diagnostic and Interventional Neuroradiology, University of Bern, Bern, Switzerland; 6 ARTORG Center for Biomedical Engineering Research, Univ. of Bern, Bern, Switzerland; 7 Chan Division of Occupational Science and Occupational Therapy, University of Southern California, Los Angeles, CA USA; 8 TranslaTUM – Central Institute for Translational Cancer Research, Technical University of Munich, Munich, Germany; 9 Helmholtz AI, Helmholtz Zentrum Munich, Munich, Germany; 10 Department of Quantitative Biomedicine, University of Zurich, Zurich, Switzerland; 11 Department of Neurology, Klinikum rechts der Isar, School of Medicine, Technical University of Munich, Munich, Germany

**Keywords:** Machine learning, Stroke, Learning algorithms

## Abstract

Magnetic resonance imaging (MRI) is an important imaging modality in stroke. Computer based automated medical image processing is increasingly finding its way into clinical routine. The Ischemic Stroke Lesion Segmentation (ISLES) challenge is a continuous effort to develop and identify benchmark methods for acute and sub-acute ischemic stroke lesion segmentation. Here we introduce an expert-annotated, multicenter MRI dataset for segmentation of acute to subacute stroke lesions (10.5281/zenodo.7153326). This dataset comprises 400 multi-vendor MRI cases with high variability in stroke lesion size, quantity and location. It is split into a training dataset of n = 250 and a test dataset of n = 150. All training data is publicly available. The test dataset will be used for model validation only and will not be released to the public. This dataset serves as the foundation of the ISLES 2022 challenge (https://www.isles-challenge.org/) with the goal of finding algorithmic methods to enable the development and benchmarking of automatic, robust and accurate segmentation methods for ischemic stroke.

## Background & Summary

Stroke is a leading cause of morbidity and mortality worldwide^1^. Up to two thirds of stroke survivors suffer permanent disability^2^. In the last decade, the advent of endovascular reperfusion therapy has significantly improved stroke outcome in patients with large vessel occlusions^3–6^. Image-based guidance of revascularization treatment decisions has further improved patient outcome for computer tomography (CT)^7–9^ and magnetic resonance imaging (MRI)^10,11^. Computer aided image analysis, especially for CT perfusion data has already found entry into clinical routine in many centers and is recommended by national guidelines^12^ to aid decision making regarding reperfusion therapy^13–16^. Machine learning and deep learning approaches have been shown to facilitate clinical interpretation of CT perfusion data and have been widely adopted in clinical routine^17–22^. Segmentation based volumetric analyses of stroke lesions in magnetic resonance imaging (MRI) are often performed for research purposes and have been shown to predict clinical outcome^23–26^. However, stroke lesion segmentations are usually painstakingly performed by hand and the quality of annotations are heavily dependent on preexisting neuroimaging experience of the rater and the total time and effort invested. The time-consuming nature of this task prevents the regular use of segmentations during clinical routine, which is further impeded by a high inter-observer variability. Automated annotation of stroke lesions could be used in clinical routine to guide therapeutic decisions in an acute setting and to predict outcome at the subacute to chronic stage. Stroke lesion segmentation could also be used to automatically classify stroke etiology in post-stroke MRI.

The first Ischemic Stroke Lesion Segmentation (ISLES) challenge, which took place in 2015, was split into two sub-challenges: Sub-acute Stroke Lesion Segmentation (SISS) and Stroke Perfusion Estimation (SPES). The goal of SISS (with a total of 64 cases for training and testing) was to segment subacute stroke lesions using conventional post-stroke MRI sequences, including T2 and T1 weighted imaging, fluid attenuated inversion recover (FLAIR), and DWI^27^. The ISLES challenge 2018, which was the previous challenge edition, was set up to predict infarct core delineated in diffusion weighted imaging (DWI) using CT perfusion data^21^. Both ISLES events received major attention from the research community: there were 120 database downloads until the ISLES15 challenge day with 14 participating teams, and the number of participating teams was roughly duplicated in the latest ISLES'18 edition. The ISLES'15 and ISLES'18 challenges played a crucial role in identifying prominent methods for acute and sub-acute ischemic stroke lesion segmentation. These datasets have since served as important benchmarks for the scientific community.

Based on the experience gained from these previous editions, ISLES'22 aims to benchmark acute and sub-acute ischemic stroke MRI segmentation using 400 cases, a more than 6-fold increase in numbers compared to ISLES15. This dataset is provided to train and benchmark DWI infarct segmentation in acute and sub-acute stroke. ISLES'22 differs in several ways from the previous challenge editions in ischemic stroke by: 1) targeting the delineation of not only large infarct lesions, but also of multiple embolic and/or cortical infarcts (typically seen after mechanical recanalization), 2) by evaluating both pre- and post- interventional MRI images in a multicenter and multi-scanner dataset, 3) generalizability of the models will be tested in a hidden dataset which also includes cases from a center that the algorithms were not exposed to in the training stage. For detailed information about the ISLES'22 challenge event, readers are referred to^28^. This challenge, together with the ATLAS challenge (https://atlas.grand-challenge.org/)^29^ are using the web-based platforms http://www.isles-challenge.org/ and https://isles22.grand-challenge.org/.

We chose to include all imaging sequences relevant for the radiological diagnosis of acute to subacute stroke lesions in MRI (FLAIR, DWI and ADC). The information contained in these imaging sequences allow a secure diagnosis of infarct and differentiation of infarcts from other cerebral lesions like older glial scars (gliosis, this may be due to a range of differing pathologies) or artefacts in DWI (e.g. scull base artefacts). The inclusion of all three imaging parameters also allow a precise spatial differentiation of infarct vs. healthy brain tissue. In the parallel held ATLAS challenge^29^, segmentation is performed solely on T1 in the chronic stage of stroke, were infarcts are reduced to a glial scar. In this setting, it is much more difficult to accurately segment the infarct borders and to visualize smaller infarcts. Additionally, it is difficult to accurately determine the etiology of the gliosis (e.g. infarct vs. trauma or bleeding).

In the challenge for the here described dataset, teams will deal with a wider ischemic stroke disease spectrum, involving variable lesion size and burden, complex infarct patterns and variable anatomical lesion location in data from multiple centers. The diversity of the ISLES'22 dataset will provide a unique challenge for participants.

## Methods

### Ethical statement

This retrospective evaluation of imaging data was approved by the local ethics boards of all participating centers. Requirement of written informed consent was waived by all ethics boards due to the retrospective nature of the study and the rigorous patient de-identification of the data.

### Subject selection

Inclusion criteria for the dataset: Subjects 18 years or older who had received MR imaging of the brain for previously diagnosed or suspected stroke were included in this study. The imaging protocol required at least a FLAIR and DWI sequence. DWI consists of a trace image at a b-value up to 1000 s/mm² as a well as its corresponding apparent diffusion coefficient (ADC) map. Image acquisition was performed on one of the following devices: 3 T Philips MRI scanners (Achieva, Ingenia), 3 T Siemens MRI scanner (Verio) or 1.5 T Siemens MAGNETOM MRI scanners (Avanto, Aera). MRIs included were intentionally chosen to be heterogeneous to ensure the best possible training and generalization of the algorithms. See Table 1 for an overview of MRI acquisition parameters by center. Images were obtained by healthcare professionals as part of the clinical imaging routine for stroke patients at three different stroke centers.

**Table 1:** Overview of MR imaging parameters.

Center	Modality	TR (ms)	TE (ms)	TI (ms)	Flip Angle (°)	Voxel (mm2)	Slice thickness (mm)	Gap (mm)
#1	FLAIR	[4800–12000]	[120–395]	[1650–2850]	[90–180]	[0.23 × 0.23–1.00 × 1.00]	[0.68–5.00]	[0.71–5.00]
	DWI	[3175–16439	[55–91]	—	[90–90]	[0.87 × 0.87–2.00 × 2.00]	[2.00–5.00]	[2.00–5.00]
#2	FLAIR	[8500–8500]	[82–111]	[2440–2440]	[150–150]	[0.43 × 0.43–0.86 × 0.86]	[4.80–9.60]	[4.80–6.00]
	DWI	[3500–4780]	[67–89]	—	[90–180]	[0.57 × 0.57–2.00 × 2.00]	[3.00–6.50]	[4.80–4.80]
#3	FLAIR	[6500–8000]	[103–104]	[2200–2370]	[150–150]	[0.45 × 0.45–0.45 × 0.45]	[6.50–6.50]	[6.5–6.5]
	DWI	[13000–13000]	[90–90]	—	[90–90]	[0.89 × 0.89–1.87 × 1.87]	[2.00–6.50]	[0.00–0.00]
All	FLAIR	[4800–12000]	[82–395]	[1650–2850]	[90–180]	[0.23 × 0.23–1.00 × 1.00]	[0.68–9.60]	[0.71–6.50]
	DWI	[3175–16439]	[55–91]	—	[90–180]	[0.57 × 0.57–2.00 × 2.00]	[2.00–6.50]	[2.00–5.00]

**Fig. 1:**
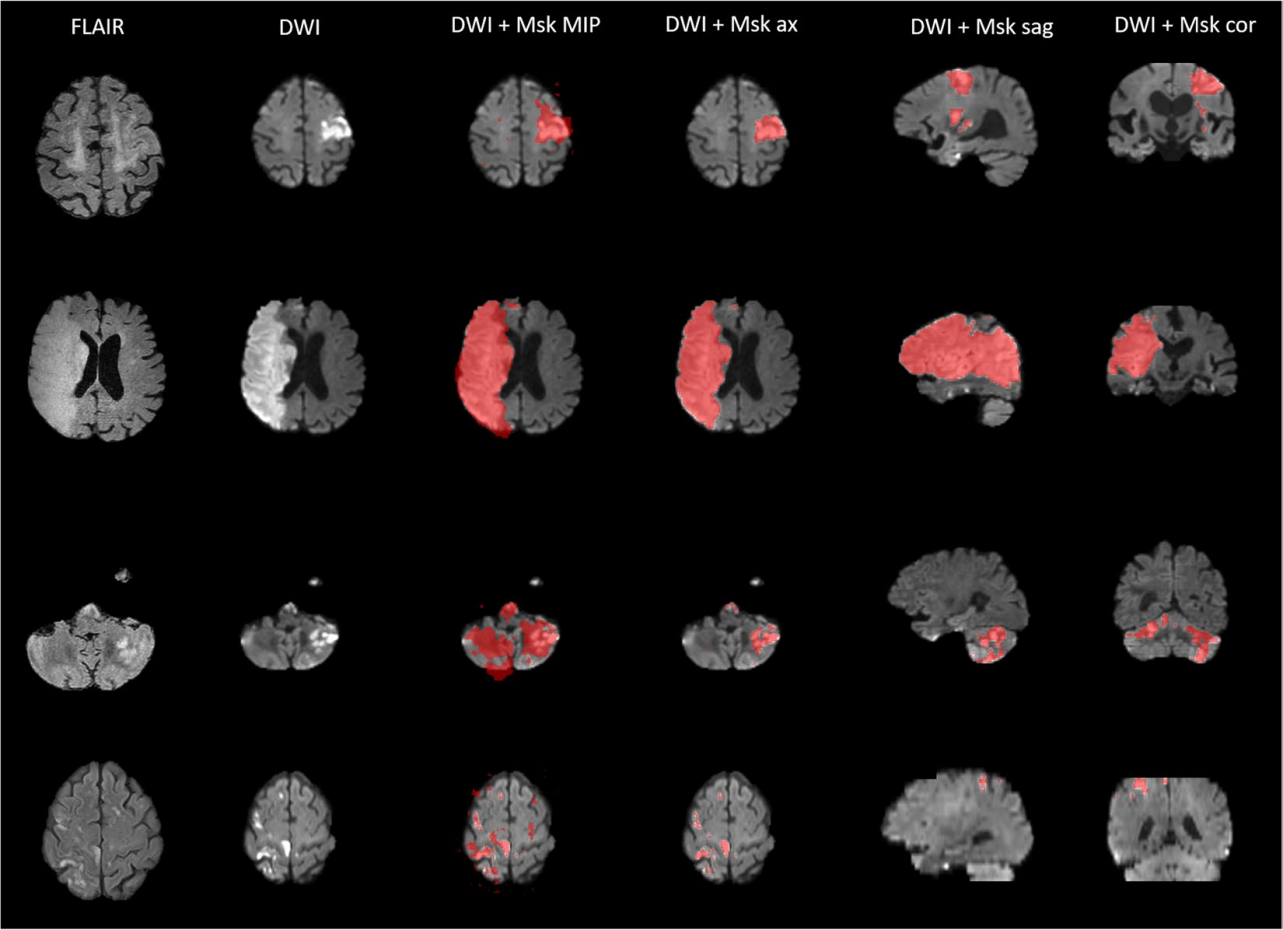
Exemplary 3D Snapshots through the infarct center of mass. Axial FLAIR and DWI images are displayed in the tow leftmost columns. The 3^rd^ colum from the left shows the maximum intensity projection (MIP) of the mask (Msk). The three rightmost columns show DWI with mask overlay (without MIP) in the three anatomical planes. Top row: Larger left-sided infarct with bilateral punctiform, likely embolic satellite ischemias. 2^nd^ row: Infarct of the entire right sided middle cerebral artery territory. 3^rd^ row: Bilateral cerebellar and occipital infarcts after posterior circulation ischemia. Bottom row: Bilateral punctiform infarcts, likely resulting from multiple micro-embolic occlusions.

Care was taken to select a broad spectrum of infarct patterns. All vascular territories were included at a similar rate. A large subset of patients with posterior circulation infarct (e.g. due to basilar artery occlusion) was included in this study. Due to a high degree of closely situated scull-base artefacts in DWI, infratentorial infarcts are difficult to segment for unexperienced raters and are frequently overlooked by segmentation algorithms with little training exposure to posterior circulation infarct. These additional labeling challenges lead us to include more cases of posterior circulation ischemia than would be expected if case selection were truly random. Figure 1 shows sample cases portraying the infarct spectrum included in this dataset.

Segmentation difficulties also frequently arise in cases with large amounts of punctiform infarcts. For human raters, to adequately capture the entire infarct territory, a time-consuming segmentation effort is required. Similarly, machine trained algorithms frequently fail to capture all the small affected regions. In n = 3 patients in the training dataset, where MR imaging was acquired for suspected stroke, no infarct was found. We chose to include this small subset to diversify the dataset. Table 2 gives an overview of infarct volumes per scan (scan infarct volume) and per unconnected lesion (lesion-wise infarct volume) for each center, as well as the number of unconnected infarcts per scan in the entire dataset. The number of unconnected infarcts was calculated using the python library cc3d^30^.

**Table 2:** Summary infarct lesion statistics for the ISLES 2022 dataset.

Center	Statistic	Scan infarct volume (ml)	Number of unconnected infarcts	Lesion-wise infarct volume (ml)
#1	Mean (std)	27.15 (51.79)	8.21 (8.33)	3.31 (19.39)
	[Min, Max]	[0.00, 482.15]	[0, 46]	[0.01, 477.26]
#2	Mean (std)	17.45 (24.55)	11.57 (19.10)	1.51 (8.30)
	[Min, Max]	[0.00, 163.97]	[0, 126]	[0.01, 163.90]
#3	Mean (std)	40.77 (46.80)	8.58 (7.62)	4.75 (20.12)
	[Min, Max]	[0.23, 233.85]	[1, 37]	[0.01, 233.62]
All	Mean (std)	26.38 (46.23)	9.11 (12.06)	2.90 (16.77)
	[Min, Max]	[0.00, 482.15]	[0, 126]	[0.01, 477.26]

Min: minimum; Max: maximum; std: standard deviation.

In the hyper-acute phase of ischemic stroke, up to 4.5 hours post onset, restricted diffusion is present (high signal on DWI and low signal on ADC) while the FLAIR in the affected parenchyma may remain without changes. This imaging phenomenon is called a FLAIR-DWI mismatch and is used in clinical practice to estimate the time window in patients where the time of onset is unknown. An accurate estimate of the time of onset is crucial to make decisions regarding revascularization treatment^11^. In acute ischemic stroke, following this hyper-acute phase and usually defined in literature as 0 to 7 days from onset, DWI and FLAIR show a high signal with reduced ADC values in the affected brain parenchyma. In the subacute stage, between 1 to 3 weeks post onset, high DWI signal begins to diminish while ADC first normalizes to values of healthy brain tissue, a phenomenon frequently referred to as pseudonormalization. FLAIR signal remains high. In the chronic stage, beginning 3 weeks after onset, DWI signal is variable but usually iso- to hypointense depending on underlying T2 signal, while ADC values are high^31–40^. MRIs with late acquisition post stroke (>1 week) often lead to a decreased DWI signal intensity for ischemic brain parenchyma. A lower signal intensity in DWI leads to lower MRI sensitivity for stroke and segmentation difficulty for both human and machine raters. In these cases, it is especially difficult to adequately annotate the border between infarct and healthy brain tissue. This dataset includes cases of MRIs in various stages of sub-acute stroke from multiple previous studies^41–44^ to find machine learning solutions to this frequent issue in stroke lesion segmentation.

### Ground truth stroke lesion segmentation

A hybrid human-algorithm annotation scheme was applied to segment all cases from Center #1 and #3 (data from the Technical University Munich and University Medical Center Hamburg-Eppendorf, see below). First, the MR input data was anonymized by conversion to Neuroimaging Informatics Technology Initiative (NIfTI) format (https://nifti.nimh.nih.gov/nifti-1), according to the Brain Imaging Data Structure (BIDS) convention (https://bids.neuroimaging.io/). As part of pre-processing, DWI and its corresponding ADC map were resliced using ANTs^45^ to an axial isotropic voxel size of 2 × 2 mm^2^. This was performed only for the cases of Center #1 (see below), as this represents the original acquisition voxel size. The slice thickness remained as acquired. FLAIR data remained as exported.

In most cases for stroke lesion segmentation, it is easier to edit and revise an existing annotation than to create an annotation ‘from scratch’. Therefore, under consideration of the caseload in this challenge and in contrast to previous iterations of the ISLES challenge, a pre-segmentation algorithm (3D UNet^46^) was trained on DWI data stemming from the Technical University Munich (Center #1, see below) that was previously annotated for other research projects. This algorithm was trained solely using a single MRI modality (B = 1000 DWI). Manually pre-segmented data intended for the training of this algorithm underwent rigorous quality control. Annotations with suboptimal quality were frequent; these cases were edited by a neuroradiology resident and checked by a senior neuroradiologists before being admitted to the training of our pre-segmentation algorithm. These cases were also included in the ISLES challenge.

**Fig. 2:**
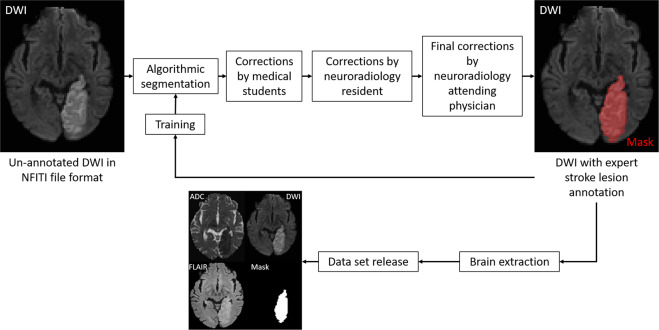
Workflow graphic of the hybrid human-algorithm stroke lesion segmentation applied in this dataset.

This house-trained algorithm later pre-segmented all thus far un-annotated scans intended for later release. These algorithmic segmentations were then checked and edited by medical students with special stroke lesion segmentation training. The pre-segmentation algorithm was updated once as more high-quality segmentations became available for training. This resulted in more accurate predictions and a lesser effort of correcting annotations by medical students. All medical-student edited annotations were critically revised and further edited by the neuroradiologist in training and the final data sets were reviewed and approved by one out of three attending neuroradiologists, all of them with more than 10 years of experience in stroke imaging. If algorithmic segmentation was deemed insufficient, which sometimes occurred in the first version of the pre-segmentation algorithm, the medical students could discard the algorithmically generated mask and manually annotate ‘from scratch’. The later instances of quality control (resident and senior physicians) were blinded to the method of pre-segmentation (algorithmic followed by student changes vs. ‘from scratch’ student segmentation), lowering the possibility of a resulting annotation bias. Manual stroke lesion segmentations were performed using ITK-Snap^47^ or 3D Slicer^48^ both open-source tools for brain imaging visualization and segmentation. Figure 2 shows an overview of annotation work-flow in preparation of the release of the dataset.

Although the pre-segmentation algorithm was trained only using a single MRI modality (B = 1000 DWI) for reasons of convenience, the final expert annotation includes in the information of all three later released modalities (DWI, ADC, FLAIR). Stroke lesion identification in clinical practice is always performed under consideration of all three released modalities. DWI is reviewed as a primary stroke imaging sequence. If the reader is unsure of the authenticity of a stroke lesion (e.g. due to suspected DWI artefact at the scull base or to differentiate T2 ‘shine-through’ from true diffusion restriction), ADC and FLAIR are reviewed. All three sequences are therefore essential for accurate classification of stroke lesions. As in clinical practice, the expert raters annotating this dataset reviewed ADC and FLAIR in addition to DWI in order to achieve the best possible annotation. For this reason, readers aiming to use this dataset for the generation of segmentation algorithms are encouraged to integrate all three sequences into their approach in order to achieve the best possible result.

### Post-annotation data pre-processing

In preparation for data-set release and in accordance to the ethical approval obtained for this challenge, the imaging data was irreversibly anonymized. For this de-identification of patients, brain-extraction was performed mask-based using the HD-BET algorithm^49^ completion of the annotation process. FLAIR to DWI rigid registration was performed using Elastix^50,51^ and subsequent skull-stripping of DWI and ADC using the registered brain mask was performed. After skull stripping for de-identification, all imaging sequences were returned to their native space before data release.

### Inter-rater analysis

In order to understand the impact of different expert annotations over the segmented stroke lesions, an inter-rater delineation experiment was conducted. In this experiment, 10 cases from ISLES’22 were selected and re-delineated by two expert neuroradiologists with more than 16 (rater II, JSK) and 10 (rater III, BW) years of experience in the field. The cases were chosen as variable as possible, including large and small infarcts, large vessel occlusion strokes and embolic pattern lesions, located in different anatomical brain areas. Dice coefficient was computed as a metric of delineation overlaps across raters. The lesion volume differences across raters were also included. The inter-rater results are summarized in Table 3.

**Table 3:** Inter-rater analysis.

	ISLES vs Rater II		ISLES vs Rater III		Rater II vs Rater III	
	Dice	VD (ml)	Dice	VD (ml)	Dice	VD (ml)
Mean (std)	0.90 (0.09)	2.37 (2.59)	0.86 (0.13)	6.56 (13.37)	0.83 (0.12)	7.22 (11.32)
[Min, Max]	[0.69, 0.98]	[0.11, 7.34]	[0.61, 0.98]	[0.06, 45.66]	[0.57, 0.98]	[0.06, 38.32]

VD: Volume difference; Dice: Dice coefficient: Min: minimum; Max: maximum; std: standard deviation.

In reviewing annotations that were previously performed for other research projects (these were used for primary training of our pre-segmentation algorithm after expert edits as described above), generally inadequate quality of the segmentations was a frequent finding. Most frequently, the expert raters of this dataset found that the infarct border was frequently traced too conservatively, leading to underestimation of the infarct volume. DWI decreases gradually in signal along the border of the infarct due to partial volume effects and thus, in order to accurately segment the edges, it is often helpful to review the high resolution FLAIR image, where infarct edema can be visualized and healthy tissue can be sharply differentiated from the infarct. Another frequent issue with segmentation, as discussed above, is the massively time-consuming effort required to accurately annotate a large number of punctiform infarcts resulting from an embolic shower. As this infarct pattern results frequently in patients who underwent successful mechanical thrombectomy, it frequently occurred within this dataset. Among the preexisting segmentations, we found that large quantities of small embolic infarcts were almost always inadequately annotated, further highlighting the need for an accurate algorithmic solution to stroke lesion segmentation.

The ground truth in this dataset, just like in any human-annotated dataset, is only as good as the performance of the human raters responsible for the annotation. With a special focus on the difficulties mentioned above, annotations were performed using a multi-layered human-algorithm hybrid workflow to distribute the work-load amongst the raters and to maximize overall segmentation accuracy. As is evident in Table 3, the annotations within the released dataset had higher Dice score and a lower volume difference with the individual expert raters than the expert raters amongst themselves.

### Probabilistic lesion map of ISLES’22

**Fig. 3:**
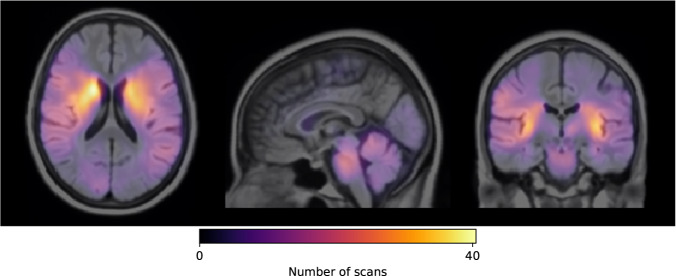
ISLES’22 probabilistic lesion map overlapped to an MNI FLAIR template.

A 3D, probabilistic lesion map was generated in order to understand the voxel-wise lesion distribution of the entire dataset (see Fig. 3). With this aim, all DWI scans from the ISLES’22 train and test datasets were linearly registered to a FLAIR MNI template (Schirmer *et al*.)^52^ using NiftyReg (Ourselin *et al*.)^53^. Suboptimally registered images were identified through visual quality control and were later re-registered until achieving an appropriate alignment with the atlas. Later on, the lesions masks were projected to the MNI space using the DWI to FLAIR found registration matrices. The probabilistic atlas was finally obtained by counting, voxelwise, the number of scans that showed overlapped infarcted tissue. The 3D probabilistic lesion map is available in Nifty format through Zenodo (https://zenodo.org/record/7335305)^54^. Sample snapshots of the probabilistic lesion map registered to an MNI FLAIR template are shown in Fig. 3.

Furthermore, we have quantified the spatial distribution of the stroke lesions according to the vascular territories irrigated by the middle, anterior, posterior cerebral arteries as well as by the infratentorial vasculature (including the pons, medulla and cerebellum areas). The spatial distribution of the lesions was computed by calculating the center of mass of the MNI-registered stroke lesions and by overlapping them to a vascular territory atlas (Schirmer *et al*.^52^, 10.5281/zenodo.7335305)^54^, see Table 4.

**Table 4:** ISLES’22 spatial lesion distribution across vascular territories.

Dataset	Vascular territory			
	ACA	MCA	PCA	Infratentorial vasculature Pons / Medulla / Cerebellum
Train	15 (6.1%)	129 (52.2%)	26 (10.5%)	77 (31.2%)
Test	6 (4.1%)	93 (62.8%)	18 (12.2%)	31 (20.9%)
All	21 (5.3%)	222 (56.2%)	44 (11.1%)	107 (27.1%)

The number of scans (percentage) is shown. ACA: Anterior cerebral arteries; MCA: middle cerebral arteries; PCA: posterior cerebral arteries.

### Challenge metrics

The evaluation metrics for results submitted by competing teams in the ISLES challenge were chosen to evaluate performance of 1) correct segmentation of large lesions, especially their borders and 2) detection of most, if not all small punctiform infarcts. Therefore, individual segmentation metrics (as Dice coefficient only) are unlikely to be sufficient, since small lesions may not consistently drive changes in some overlap measures (for instance, in the presence of a large stroke lesion and a very small separated embolic infarct, a large Dice increase will come by only detecting the large lesion, even if the small lesion is missed). Thus, with a focus on clinical translation, we consider metrics that are often of main interest for neuroradiologists, such as the lesion volume, the presence/absence of a lesion (i.e, detection) and the accurate count of the lesion burden. However, we also included classical segmentation metric as Dice similarity coefficient to gain an overall impression of the performance overlap between ground truth and predictions. The following error metrics will be used for scoring:

Dice similarity coefficients for segmentation masks and the absolute difference for infarct volume will be computed as voxel-wise surrogates for model performance. As a lesion wise metric the lesion count absolute difference in the predicted mask will be calculated obtained by computing the amount of connected components per case. As an additional lesion-wise metric, the lesion detection f1 score will be calculated.

As in previous iterations of the ISLES challenge, ranking is produced by comparing each metric at the case level. In short, metrics are calculated for each case, followed by establishing metric-specific rank separately for each dataset. A mean rank over all metrics is obtained to obtain the team’s rank for each case. The final rank is the mean of all case-specific ranks. Scoring will be performed on a script-automated basis.

## Data Records

### Data repository and storage

All training data (n = 250) has been made publicly available under the creative commons license CC-BY-4.0 in the preprocessed format on Zenodo (10.5281/zenodo.7153326)^55^. Further information about the ISLES challenges can be found under http://www.isles-challenge.org/ and under https://isles22.grand-challenge.org/.

### Data structure and file formats

All medical imaging files were exported from the Picture Archiving and Communication System in the NIfTI format. Segmentation masks are created and saved in NIfTI format. All data in the ISLES’22 dataset was separated into a training dataset (250 subjects) and a test dataset (150 subjects). Corresponding scanner metadata from the Digital Imaging and Communications in Medicine (DICOM) header in the JSON file format is provided with the datasets, if available. MRIs from the following centers were included:

Center #1: University Hospital of the Technical University Munich, Munich, Germany.

Center #2: University Hospital of Bern, Bern, Switzerland.

Center #3: University Medical Center Hamburg-Eppendorf, Hamburg, Germany.

The publicly available train set comprises data from centers #1 and #2. The test set comprises data from all the three centers in equal parts as follows:

Acute to early sub-acute stroke data from centers #1 and #3 (MRIs acquired after revascularization therapy).

Hyper-acute to acute stroke data from center #2 (MRIs acquired before revascularization therapy).

Thus, in this ISLES’22 task we will evaluate the robustness and generalization capability of the proposed models over 1) new scans coming from two centers already used at the training stage, 2) new scans coming from a new (unseen at training stage) center, and 3) new scans acquired before revascularization therapy, coming from a center already seen at training stage. Introduction of a previously unseen center at the test stage likely results in an overall deterioration of algorithmic performance but in turn allowing a better differentiation of the generalizability of the solutions.

The split between training and test data set has been performed so that both sets include a similar variance of stroke lesion patterns ranging from large territorial infarcts to small punctiform ischemia. The spatial distribution of lesions according to the different vascular territories is summarized in Table 4. The majority of the scans have lesions within the territories irrigated by the middle cerebral arteries and the infratentorial vasculature. Similar spatial distributions between training and test datasets have been considered. A slight difference in the distribution of infratentorial scans (~10% more cases in the train set than in the test set) and in middle cerebral arteries territory (~10% more cases in the test set than in the train set) can be observed.

## Technical Validation

The presented medical imaging data was derived from the picture archiving and communication system of the corresponding institutions and therefore fully complies with the legal standards and quality controls for the acquisition of medical imaging in Germany, the European Union and Switzerland, as well as the industrial standards of the scanner vendors. Segmentation masks were prepared and annotated at voxel-level by a human-machine hybrid algorithm with hierarchical manual checks and corrections first by specifically trained medical students and later a neuroradiology resident. Afterwards, the masks were reviewed, corrected, and finally approved by an expert neuroradiologist, ensuring a high quality standard.

We aimed to create a dataset which is representative of real world stroke cases. Therefore, only cases with massive motion artefacts, leading to images that are unusable in diagnostic clinical practice, were excluded from the dataset. We chose not to exclude cases based on other potential quality issues like signal loss or spatial distortions, relying on the imaging standards that are established in clinical practice at the participating centers. Similarly, no special regard was given to cases if they were acquired at 1.5 T as opposed to 3 T and the raters were blinded to mode of image acquisition.

## Data Availability

In order to facilitate future users of this dataset to get familiarized with the images, we have released the following ISLES 2022 Github repository: https://github.com/ezequieldlrosa/isles22. The repository contains scripts to read the images, visualize them, and to quantify the algorithmic results performance with the same metrics used in the challenge to rank participants. Besides, we have also released the image container that is used during the challenge in order to evaluate the participants’ algorithm submissions (https://github.com/ezequieldlrosa/isles22_docker_evaluation).
